# An intergenerational programme delays health impairment in nursing home residents: the Duplo project

**DOI:** 10.1007/s41999-022-00700-x

**Published:** 2022-10-17

**Authors:** Inmaculada Boyano, Sonia Nieto, Jose-Antonio Serra, Marisa Alcaide, Marta Caparros, Manuel Varela

**Affiliations:** 1 Geriatric Department, Hospital Universitario de Mostoles, Madrid, Spain; 2 Universidad Francisco de Vitoria, Madrid, Spain; 3 Geriatric Department, Hospital General Universitario Gregorio Marañón, Madrid, Spain; 4 Biomedical Research Networking Centre on Frailty and Healthy Ageing, CIBERFES, Madrid, Spain; 5 School of Medicine, Universidad Complutense, Madrid, Spain; 6 Fundacion Gregal, Madrid, Spain; 7 Department of Internal Medicine, Hospital Universitario de Mostoles, Madrid, Spain

**Keywords:** Intergenerational programmes, Cognitive impairment, Functional impairment, Nursing homes

## Abstract

**Purpose:**

To analyse whether an intergenerational programme in which students interacted with institutionalised older persons had any impact on the older persons’ functional status.

**Methods:**

Each academic year, a group of older adults living in nursing homes were divided into two arms. For the next four months, the first group received daily visits from a group of students during which they followed a pre-established activity plan, whilst the other arm proceeded with their normal activity. After 4 months, the groups crossed over, and the second arm received the student visits, whilst the first group returned to their normal activity.

A battery of tests was performed at inception, crossover and the end of the second period. The tests explored mobility (Timed Up-and-Go), cognition (Mini-Mental Examination), executive function (Frontal Assessment Battery) and mood (Geriatric Depression Scale). A dichotomous aggregate “significant impairment” variable was deemed to be present when there was at least a 20% loss of function (compared to the value at the beginning of the period) in any of the aforementioned tests.

**Results:**

The study included 289 older adults who visited with 91 students. Subjects in the active phase had a lower incidence of significant impairment than those in the control phase (O.R. 0.90, *p* < 0.01). There were no significant differences in the individual variables.

**Conclusion:**

An intergenerational project with students visiting older adults in nursing homes had a protective effect, delaying functional decay in older adults.

## Key summary points

**Aim:**

To evaluate whether an intergenerational programme in which students visit institutionalised older adults following a pre-established activity agenda has beneficial effects on residents’ health status.

**Findings:**

An aggregate “significant impairment” variable was defined as a loss of at least 20% of the baseline values in any of four variables: mobility, cognitive function, executive tasks and mood. The programme reduced the incidence of significant impairment (odds ratio 0.9, *p* < 0.01).

**Message:**

Bringing together students and institutionalised older adults delays functional decline.

## Introduction

### Background

In most Western countries, the population is ageing. In Spain in 2018, 21% of citizens were over 65 years old, and 6% were over 80 (63% of whom were women) (INE, 2020). As our society as a whole becomes older and our life expectancy is ever rising, many older adults are institutionalised and live isolated in spaces in which they interact mostly with other older adults.

Although ageing is an individual process, it is highly influenced by long-standing structural elements, and amongst them, social networks are an extremely important factor [1, 2]. Social support promotes healthy behaviours and helps in coping with stress and disease [3, 4], whilst loneliness is a significant factor influencing older adults’ health status [5, 6]

Several pathologies prevalent in older adults are associated with dependency and disability and often result in more or less indefinite institutionalisation. The most frequent causes of institutionalisation in the Spanish context are functional impairment after hospital admission, hip fracture, dementia, stroke, advanced chronic disease or the loss of a spouse. In Spain, 30% of the older adults admitted to nursing homes are between 85 and 89 years old, and the proportion of the oldest aged amongst nursing home residents has been increasing since 2011 [7]. Furthermore, institutionalised older adults are more vulnerable both in physical and cognitive terms and have fewer social networks than persons living at home [8].

Concurrently with this ageing/isolating process, young generations hardly connect with the ageing population. A recent survey [9] showed that 75% of young adults did not share their lives with people over 65 years old and that only 13% were satisfied with their relationship with their grandparents. Furthermore, 41% of the people interviewed believed that young people show indifference and lack of interest towards older adults [9].

Intergenerational programmes (IPs) are interventions designed to increase interaction, cooperation and exchange between people of different generations, with the aim of benefitting both sides. On the one hand, they help older adults continue to be productive and appreciated members of the community. In addition, they build bonds between people of different ages and help transmit traditions and values to other generations, improving older adults’ self-esteem. On the other hand, young adults receive direct experience with a reality that surrounds us (even if we turn our backs to it). Older adults contribute to young adults’ life experiences and may provide new, more mature points of view on life and its quandaries. Whilst interacting with older adults, young adults may obtain new insights, improve their social skills and change their perception of ageing and elderly people. Furthermore, this experience could help them face the hard fact of ageing, both for themselves and their relatives, and help them eventually avoid certain ageist stereotypes.

There are numerous publications reporting on the positive effects of IPs on satisfaction and life quality, both in young and older adults [3, 4, 10–14]. Intergenerational contact improves generativity as well as physical and intellectual function whilst reducing or slowing cognitive and functional decline, improving depressive symptoms and reducing the risk of loneliness and isolation [15–18].

However, IPs are extremely diverse [4], both in terms of the target population (infants, adolescents, frail or impaired older adults or healthy older adults living at home), the setting (older adults visiting schools, students visiting nursing homes), the activities (reading, playing, helping) and the type of interaction (face-to-face, remote). IPs are generally a social intervention rather than an experiment, and thus, their results may not be easily extrapolated. Furthermore, they are usually analysed on social and psychological grounds rather than based on hard medical data.

### Purpose of the Duplo programme

In 2016, a private foundation (https://fundaciongregal.org/) launched a project (https://fundaciongregal.org/programa-duplo/) to promote intergenerational contact between institutionalised elderly people and young students.

This project could arguably benefit both groups. For older adults, it could provide first-hand contact with a reality out of their scope through interaction with young people whilst arguably improving their self-esteem, as they could contribute to their life experiences and insights. Furthermore, the intervention could increase their motivation and activities, boost their social lives through conversation and other social activities and hopefully improve (or slow the loss of) functionality as well as promote active ageing.

For young students, this interaction could make them aware of their potential contribution to society, provide new perceptions of age and ageing and improve their social skills. Furthermore, the students receive a scholarship whilst within the Duplo programme.

Simultaneously, a research programme was initiated to objectively explore the effects of this intervention in both older adults and students. Concerning older people, the impact of the programme on global health status was evaluated by analysing changes in well-established tests assessing physical, cognitive, executive and mood spheres. For the students, the project investigated the extent to which the programme modified their knowledge and attitudes towards ageing and older people.

The present manuscript reports on the results concerning the institutionalised population [although a brief note on the students is included in the Discussion].

## Methods

### Design

The design was a case–control, crossover observational study.

The intervention lasted one academic year (two consecutive 4 month periods) and had an active group and a control group, which were switched over in the midterm.

The programme consisted of a 4-month agenda (see “Procedure: activity agenda”) for the active group, whilst the control group followed with their normal activities. Then, there was a crossover, and the control group received the agenda whilst the first group resumed their normal activities.

A battery of tests was administered to both groups at admission, at the crossover point and at the end of the intervention (baseline, crossover and final). All tests were conducted either by one of the authors (MC) or by the registered psychologists in the nursing homes, following a standardised protocol.

For the students, a battery of tests was administered at admission and at the end of the intervention. All tests were conducted by one of the authors (MC).

The project was approved by the University Hospital of Mostoles Ethical Committee. Written informed consent was obtained from each participant or his or her relative.

### Participants

The programme was offered to all persons admitted to eight nursing homes in the urban area of Madrid (Spain) with the following inclusion criteria:

- Admitted for at least 8 weeks before inclusion
- Aged over 65

Exclusion criteria were as follows:

- Cognitive impairment as indicated by a score on the Spanish version of the Mini-Mental Examination < 16.

We initially considered excluding wheelchair-bound patients to optimise physical activities. However, this was later considered an unfair limitation, and this exclusion criterion was dropped.

**Fig. 1:**
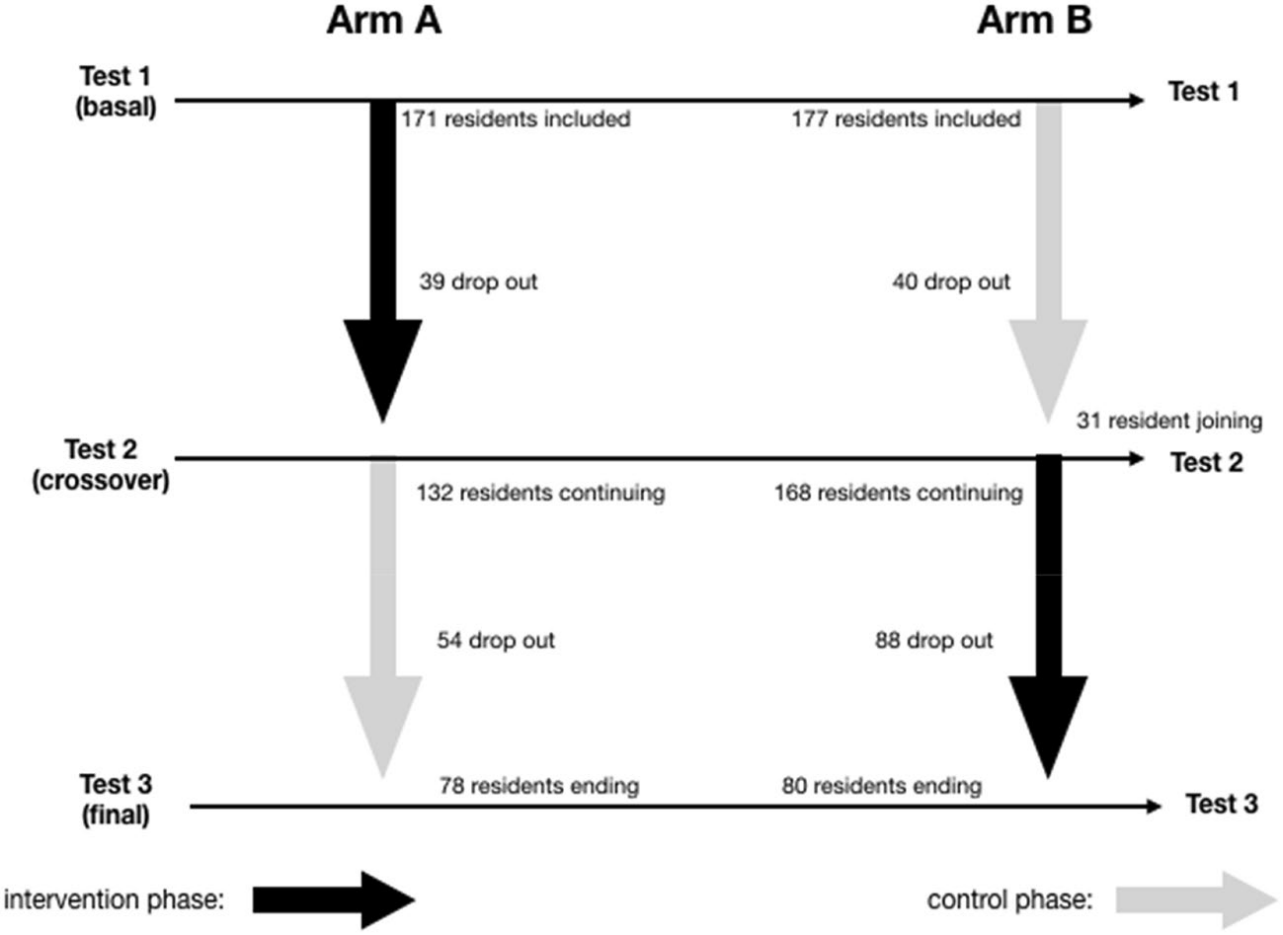
Flow chart

Figure 1 displays the flowchart of included subjects and those who dropped out.

### Measures

The research aimed at a global assessment of health status involving physical, cognitive, executive and affective aspects. The tests used to evaluate these features were chosen because of their objectivity, standardisation and wide use amongst geriatric patients.

We analysed four individual dimensions and established as the main outcome a dichotomous “significant impairment” variable, defined as present whenever the subject experienced a loss of a predefined value in any of the baseline individual dimensions during the period under analysis.

The basic dimensions were as follows:

1. Mobility, assessed by means of the Timed Up-and-Go test (TUG) [19, 20]
2. Global cognitive (mainly memory) abilities, assessed through the Spanish version of Mini-Mental Examination (Mini examen cognitivo de Lobo MEC-35) (MME) [21, 22]. Range: 0–35.
3. Frontal (mainly executive) functions, evaluated through the Frontal Assessment Battery (FAB) [23, 24]. Range: 0–18.
4. Affective/mood status, estimated by means of the Geriatric Depression Scale 15 (GDS) [25, 26]. Range: 0–15.

Finally, we included a global “significant impairment” compound variable (S.I.), with the aim of assessing if there had been relevant changes in any of the aforementioned measurements. "Significant impairment" was evaluated at the end of both the active and control phases. It was deemed to be present if any of the measurements was less than 80% of its value at the beginning of the phase (i.e. there was at least a 20% loss of function). This 80% threshold is obviously arbitrary, and therefore, we explored the behaviour of this variable when varying the S.I., defining the threshold from 10 to 90%.

### Students

The Duplo programme offered scholarships to students admitted to a public university (https://www.uc3m.es/inicio), a public vocational school (https://www.educa2.madrid.org/web/centro.ies.alarnes.getafe) and a private foundation supporting children at risk of social exclusion (https://fundacionbalia.org/).

A public call was published in each centre. Applying students were then selected based on the following criteria:

1. Academic scores (average grade)
2. Receipt of a scholarship from the Spanish Ministry of Education (implying an unfavourable economic status)
3. Provision of a motivational letter explaining his or her reasons and inspirations to participate in the programme, as well as his or her interests and hobbies
4. Completion of a battery of tests from which a team of psychologists evaluated his or her social, interpersonal and communicative skills; pro-social as well as artistic and sports backgrounds were also considered.

The selected students were committed to visit the assigned nursing home for 6 h every week following a pre-established timetable.

The student’s mean age was 21 years (sd. 2.3). Seventy-seven per cent attended a public university, 22% were completing professional training, and 1% had only elementary studies. Before starting the intervention, the students completed a 10 h training period conducted by specialised staff. This included a short introduction to the physiology of ageing, some notions on group dynamics, music therapy, art therapy, yoga and relaxation techniques, and a bringing-together session with Duplo students of the previous year.

### Procedure: activity agenda

The Duplo programme aims to provide activities that disrupt the everyday routine, emphasising areas such as the following:

- Talks and debates on subjects of interest (e.g. actual events, geography, history, art, sports, new technologies, politics)
- Walks
- Art therapy, music and artistic activities allowing the expression and sharing of mood states
- Games (cards, team games, etc.)
- Yoga and relaxation
- Life stories: Whilst not formal reminiscence therapy, an important part of the programme lies in the older adults telling their histories. Each term, students are encouraged to produce, together with an older adult, a life story of that particular older adult, focussing on whatever he or she considers most relevant.

**Fig. 2:**
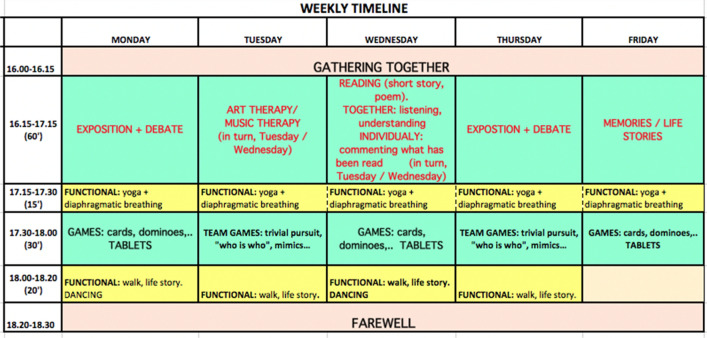
Weekly activity schedule for students and residents

Figure 2 is the weekly time line of the intervention. The nursing home residents are conveniently divided into groups of 10–15 persons, with 4 students per group. Each week, a different student is in charge of planning and managing the diverse activities performed in each nursing home, always in accordance with the pre-established schedule.

The Duplo programme in no way tries to compete with or supplant the therapist’s job in the nursing home. The students do not have a health sciences background and are not assistance staff. At all times in each nursing house, there are institutional personnel in charge whom the students may consult if necessary.

### Data analysis

We compared changes in performance (measured as impairment: (post–pre) for the TUG and GDS and (pre–post) for the MME and FAB) both in the active and control phases in each subject. These differences (post–pre in the TUG and GDS vs. pre-post in the MME and FAB) are due to the fact that an impairment translates into an increase in the TUG and GDS scores and a decrease in the MME and FAB scores.

Individual variables were tested by means of multivariate ANOVA. Significance was assumed if double-tailed *p* < 0.05. Although methodologically questionable from a statistical point of view (multiple tests instead of multivariate analysis), we also compared the pre–post results for each individual variable (Wilcoxon test due to the non-normal distribution).

Furthermore, we assessed the occurrence of “significant impairment” (as previously defined) for each subject, both in the active and the control phases. The active vs. control phases were compared by means of a chi^2^ test. To analyse the influence of the threshold defining “significant impairment”, we explored how these results varied when changing the threshold through all integer values from 10 to 90%.

A satisfaction questionnaire was also administered to each participant at the end of the intervention, asking them to rate their experience with the programme as “completely satisfactory”, “quite satisfactory”, “neither satisfactory nor unsatisfactory” or “unsatisfactory”.

All statistical analyses were performed in R (https://rstudio.com). Both the data set and the script are available upon request.

## Results

The Duplo project is still on course (although the COVID-19 pandemic forced its temporary interruption). We report on the results of the first two and a half years of the programme (from October 2017 to January–March 2020, depending on the nursing home), with a total of 379 included institutionalised persons and 91 participating students. Some older adults and students repeated in successive years, but the repeating cases were not included in the analysis.

Of the 379 persons included, 79 dropped out after the first test and 142 dropped out after the second test (including those affected by the interruption due to the COVID-19 pandemic). Thirty-one subjects joined the programme with some delay and did not perform the baseline test. In all, there were 289 subjects who had at least two successive tests. This was the population submitted to analysis. Table 1 displays the baseline characteristics of the sample.

**Table 1:** Characteristics of the nursing home residents included in the study at inception

Variable	Median (or proportion)	Quartiles
Gender (F/M)	289/90	–
Age (years)	87	53/82/87/91/104
Elementary education (at least) (%)	91	–
Timed up-and-go (TUG) (sec.)	19	8/14/19/26/102
Unable to perform TUG (%)	34	–
Mini-mental examination	26	6/21/26/30/35
Frontal assessment battery	10	2/7/10/14/18
Geriatric depression scale	4	0/2/4/7/13

### Comparison between included subjects vs. those who dropped out

There were no significant differences in gender, age, or the TUG, MME, FAB or GDS scores between the subjects included in the analysis and those who dropped out (Appendix).

### Baseline comparison between groups (first active vs. first control)

Although we initially tried to randomise cases and controls, this became impossible for practical reasons (cases and controls swapped places in nursing homes for convenience, time schedules, etc.), and several randomisation violations were unavoidable. As a consequence, the two groups (group 1: first active, then control vs. group 2: first control, then active) were not equilibrated, with a higher % of subjects unable to perform the TUG in group 2 (Appendix).

### Comparison between the active phase vs. the control phase

There were no significant differences in the attrition rate between the active and control phases (27 vs. 26%, p: n.s.).

There were no significant differences in individual variables (impairment in the TUG, MME, FAB or GDS) between the active and control phases (ANOVA: Table 2A).

**Table 2:** Comparison of loss of function between the active and control phases

A: ANOVA with the four dimensions (mobility: TUG; cognitive: MME; executive: FAB; mood: GDS) as factors and active/control phases as dependent variables					
Variable	D.f	Sum Sq	Mean Sq	F	*p* value
Mobility: (decline in TUG)(sec.)	1	0.52	0.5220	2.079	n.s
Cognition (decline in MME)	1	0.00	0.00	0.00	n.s
Executive functions (decline in FAB)	1	0.21	0.2094	0.834	n.s
Mood (decline in GDS)	1	0.05	0.0499	0.199	n.s
Residuals	414	104.46	0.2511		

B: Comparison of individual variables (Wilcoxon test)				
	Active (median (or odds), quartiles)	Control (median (or odds), quartiles)	Wilcoxon (or Chi^2^)	*p*
Mobility:	1	1	*W* = 11,206	n.s
TUG (sec.) (post-pre)	− 35/− 1/1/5/51	− 32/− 2/1/5/33		
Odds of becoming unable to perform TUG	0.39	0.29	*χ*^2^ = 1.27	n.s
Cognition	0	0	*W* = 23,253	n.s
MME (pre-post)	− 9/− 2/0/3/14	− 10/− 2/0/2/14		
Executive functions	0	0	*W* = 21,539	n.s
FAB (pre-post)	− 11/− 2/0/1/10	− 11/− 1/0/2/12		
Mood	0	0	*W* = 22,256	n.s
GDS (post-pre)	− 9/− 1/0/1/9	− 8/− 2/0/1/11		

C: Comparison of “significant impairment”, defined as a 20% loss of function (final value < 80% of initial value) in any of the four dimensions				
	Active	Control	*χ*^2^	*p* value
Odds of significant impairment	1.73	1.89	9.7	< 0.01

*TUG* Timed Up-and-Go test, *MME* Mini-Mental Examination, *FAB* Frontal Assessment Battery, *GDS* Geriatric Depression Scale

Although methodologically questionable (multiple tests instead of a single contrast), for the sake of clarity, we also performed Wilcoxon’s paired test for each individual variable. Table 2B displays the change in each dimension for the active and control groups.

Although no statistically significant differences were observed in the individual variables, there were differences in the compound “significant impairment” variable, exploring the presence of a loss of function in any of the individual dimensions. The active phase had a protective effect (odds ratio 0.91, *p* < 0.01 for a significant impairment-defining threshold of 80% of the baseline value in any of the aforementioned variables) (Table 2C).

The incidence of “significant impairment” did not differ between genders (odds of S.I. (80% threshold): women 1.79, men 1.75, *χ*^2^ < 0.1, *p* = n.s.), was not significantly affected by age (median age both for S.I. + and S.I.: 87 years old; Wilcox = 22,130, *p* = n.s.) and did not differ between groups (odds of S.I. in group 1 (first active, then control): 1.82; odds in group 2 (first control, then active): 1.74, *χ*^2^ = 0.02, *p* = n.s.)

**Fig. 3:**
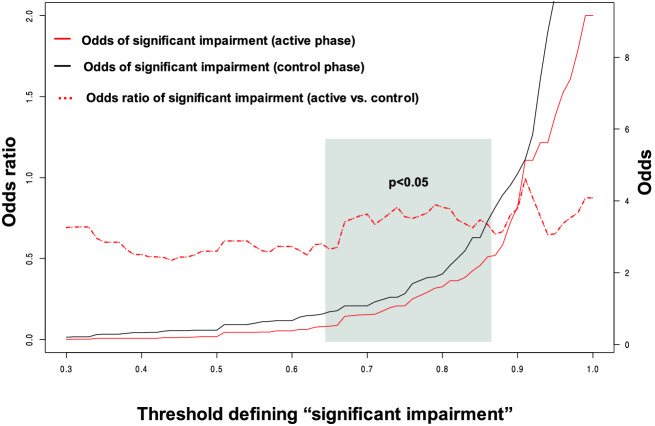
Differences in “significant impairment” between active and control phases, depending on the defining threshold (% of function in any variable (TUG, MME, FAB, GDS) with respect to values at the beginning of the phase). Red line (right axis): odds of “significant impairment” (SI) in the active phase (individuals with SI in active phase/individuals without SI in the active phase; for each SI-defining threshold). Black line (right axis): odds of “significant impairment” (SI) in the control phase (individuals with SI in control phase/individuals without SI in the control phase; for each SI-defining threshold). Red dotted line (left axis): odds ratio (odds of SI in active phase/odds of SI in control phase, for each SI-defining threshold). The odds ratio is consistently favourable for the active phase (OR < 1), but only reaches statistical significance with significant impairment-defining thresholds between 0.68 and 0.82

To explore the influence of the threshold defining significant impairment, we repeated the analysis, sweeping all the range of threshold values from 10 to 90% (Fig. 3). The differences were robust through all S.I. defining thresholds, with odds ratio consistently favouring the active group (OR < 1), although they only reached statistical significance when the S.I. defining threshold ranged between 65 and 85%.

### Satisfaction questionnaire

Regarding the satisfaction questionnaire, 66% of older adults rated the programme as “completely or very satisfactory”, 20% rated it as “quite satisfactory”, 12% rated it as "neither satisfactory nor unsatisfactory" and 2% rated it as “unsatisfactory”.

## Discussion

An intergenerational intervention in which students visit nursing homes following a pre-established agenda delays the decline of a composite health status variable aggregating functional, cognitive and mood aspects in institutionalised nursing home residents. Whilst the effect is not significant on each of the explored dimensions (mobility, cognition, executive function and mood), the impact is robust when considering the composite of all dimensions. We established a predefined threshold of 80% (significant impairment = loss of at least 20% of the baseline function in any of the aforementioned dimensions). With this definition, the odds ratio of deterioration was 0.90 in the active vs. control groups. However, this could be heavily biassed by the chosen threshold. To study the influence of the threshold definition, we analysed the effects of varying its value from 10 to 90%. As displayed in Fig. 2, the effect is robust, with an odds ratio consistently < 1 for the active phases, although statistical significance was restricted to thresholds ranging from 62 to 83% of the baseline values in any of the variables.

The interaction with the students was considered rewarding by most nursing home residents (86% considered the programme to be “completely”, “very” or “quite satisfactory”, whilst 14% judged it as “neither satisfactory nor unsatisfactory” or “unsatisfactory”).

Intergenerational programmes are extremely diverse and difficult to compare. The particularities of the present study include its prospective, crossover design and the use of objective, well-established health metrics as outcomes.

Under these conditions, we were able to detect a consistent effect of the programme in delaying health decline in institutionalised elderly persons.

### Students

Although not the object of the present publication and thus not included in the Results, some data on the students included may be of interest. The areas explored both at inclusion and at the end of the intervention were as follows:

- The Spanish version of Palmore's facts on ageing quiz [27, 28]
- A test on attitudes based on Morgan and Bengtson’s “Negative attributes of old age and positive potential in old age” [29]
- A test on implicit attitudes (AgeIAT) [30, 31].

Whilst the final analysis is still under way, some preliminary results may be offered.

In Morgan and Bengston’s test on attitudes, there was a significant increase in positive attitudes (pre: 25.46 (sd 2.93); post: 30.8 (sd 6.71), Wilcoxon *W* = 232, *p* < 0.001) and a significant decrease in negative attitudes (pre: 12.93 (sd 2.1), post: 10.82 (sd 4.06), Wilcoxon *W* = 1285, *p* < 0.001). Interestingly, the decrease in negative attitudes correlated with the baseline value (students with higher negative attitudes tended to change the most) (rho = 0.34, *p* = 0.01). No significant changes were observed in the Palmore knowledge on the facts of ageing or on the implicit attitude test delete.

### Limitations

Arguably, the easiest way to approach the data analysis would have been a two-way (group and period) ANOVA with repeated measures. However, this was fraught with problems. There was a high attrition rate (which was to be expected, with a median age of 87 at inclusion), the variable distribution suggested the unsuitability of parametric statistics, and the abrupt interruption of the programme due to the COVID-19 pandemic resulted in many subjects who participated in only one phase (either active or control) without crossover. This resulted in a very unbalanced ANOVA table, with many missing cells. Furthermore, if we selected only subjects who had completed the three tests (baseline, crossover and final), our sample decreased from the 389 initially included to only 135 subjects. We therefore decided to select subjects performing at least two successive tests, leaving 289 cases for analysis. This allowed us to obtain individual values of loss of function for each subject in the active and/or control phases and therefore contrast the loss in active phase vs. loss in control phase (non-paired Wilcoxon test). This choice resulted in a larger, arguably more representative sample size.

In any case, the results with only the sample performing the three tests were very similar to those obtained with our final selection (subjects performing at least successive tests). As an example, we include in the Appendix the “significant impairment” curves depending on the defining threshold (similar to Fig. 3), including only the subjects performing all three tests.

The 20% threshold defining the “significant impairment” variable is admittedly arbitrary. Whilst compound variables are routinely used in clinical studies, we have no strong reason to propose this 20% limit other than the clinical judgement that this threshold in any of the assessed domains may indeed make a difference in older adults’ lives. To minimise the arbitrariness of this decision, we explored the effects of varying this threshold (from 10 to 90%) (Fig. 3). The differences remained robust, with an odds ratio consistently < 1 for the active group, although as expected, statistical significance was reached only when the two subgroups (SI and non-SI) were not too unbalanced. When most—or very few—of the subjects experienced “significant impairment”, it was difficult to reach statistical significance.

Subjects who were first in the active group and then switched to the control were hardly “clean” controls since they could arguably carry on some protracted effects of the intervention. We, however, kept them as controls because this hypothetical “carry-on” effect, if ever, would act in favour of the null hypothesis (decreasing the influence of the active intervention). Thus, the differences observed between the active and the control branches had to overtake the hypothetical carry-on effect and therefore reinforce the evidence of the intervention’s efficacy.

The coexistence of an activity template and, at the same time, students’ freedom to modulate and manage these activities may be seen as both a strength and a limitation of the project. Whilst strict adherence to empirically based interventions may improve the efficacy of intergenerational programmes [10], our perception is that this flexibility improved the student's implication and clearly enhanced their ingenuity and spontaneity.

## Conclusion

In conclusion, an intergenerational programme bringing together institutionalised elderly persons with students through an organised, pre-established activity programme reduces or delays the development of age-associated impairment in a composite variable gauging functional, cognitive, executive and affective areas.

Furthermore, this intervention seems to modify students’ attitudes towards older adults (data not included in this study).

In times of ever increasing social isolation of older adults and selective blindness of youth on ageing issues, this type of project may address both problems synergistically. Although no economic evaluation has been made, these interventions may be not only socially desirable but perhaps even cost-effective.

## Clinical implications

- An intergenerational programme in which students interact with institutionalised older adults following a flexible but pre-established 10–12 h/week schedule is feasible and well accepted by both parties.
- No significant differences were observed in individual variables analysing functional (Timed Up-and-Go), cognitive (Mini-Mental Examination), executive (Frontal Assessment Battery) or mood (Geriatric Depression Score) variables between the active and control branches of the study.
- However, significant differences were observed in a combined variable including these four dimensions. The programme reduced or delayed the development of a dichotomous “significant impairment” variable, obtained by comparing pre vs. post results in each dimension in the active vs. control branch. Furthermore, this difference was consistent through various definitions of this variable.
- Whilst not included in this manuscript, the intervention also resulted in a positive change in students’ attitudes towards ageing.

## Baseline characteristics of adults completing or dropping out of the study

**Table:**

Variable (median (IQR))	Completed	Dropped out	W (or Chi^2^)	*p* value
Gender ratio (F/M)	3.2	3.3	(< 0.01)	n.s
Age	87 (9)	87 (8)	11,828	n.s
TUG	19 (13.1)	18 (11.7)	5431	n.s
% unable to perform TUG	31%	42%	(3.29)	0.06
MME	26 (9)	25 (8.5)	9959	n.s
FAB	10 (7)	10 (5)	9457	n.s
GDS	4 (5)	4 (6)	10,710	n.s

*TUG* Timed Up-and-Go, *MME* Mini-Mental Examination, *FAB* Frontal Assessment Battery, *GDS* Geriatric Depression Scale.

## Comparison between groups at inclusion (group 1: active → control; group 2: control → active)

**Table:**

Variable (median (IQR))	group 1	group 2	W (or Chi^2^)	*p* value
Sex (F/M)	3.0	3.3	(0.0)7	n.s
Age	88 (9)	86 (8)	11,828	n.s
TUG	18 (11.5)	20 (12.2)	4217	0.08
% unable to perform TUG	20	40	(12.04)	< 0.01
MME	27 (9)	25 (10)	10,157	0.06
FAB	12 (6.5)	10 (6)	9892	0.09
GDS	4 (5)	4 (5)	9241	n.s

Differences in “significant impairment” between active and control phases, depending on the defining threshold (% of function in any variable (TUG, MME, FAB, GDS) with respect to values at the beginning of the phase) including only subjects performing all tests. Red line (right axis): odds of “significant impairment” (SI) in the active phase (individuals with SI in active phase/individuals without SI in the active phase; for each SI-defining threshold). Black line (right axis): odds of “significant impairment” (SI) in the control phase (individuals with SI in control phase/individuals without SI in the control phase; for each SI-defining threshold). Red dotted line (left axis): odds ratio (odds of SI in active phase /odds of SI in control phase, for each SI-defining threshold).
